# Fatal Interstitial Pneumonitis During Combined Degarelix, Darolutamide, and Docetaxel Therapy for Metastatic Prostate Cancer

**DOI:** 10.7759/cureus.104971

**Published:** 2026-03-10

**Authors:** Fumihiro Ito, Koki Kobayashi, Gaku Hayashi, Shunsuke Kamijo, Takashi Fujita

**Affiliations:** 1 Urology, Gifu Prefectural Tajimi Hospital, Tajimi, JPN

**Keywords:** androgen receptor targeted therapy, docetaxel toxicity, drug-induced interstitial lung disease, fatal pulmonary toxicity, interstitial pneumonitis, metastatic prostate cancer, triplet therapy

## Abstract

Triplet therapy for metastatic hormone-sensitive prostate cancer improves survival but may rarely cause severe pulmonary toxicity. A 79-year-old man with low-volume metastatic prostate adenocarcinoma (PSA 613 ng/mL) received degarelix, darolutamide, and docetaxel. After the fifth docetaxel cycle, he developed a low-grade fever and exertional dyspnea. Chest computed tomography revealed diffuse, non-segmental bilateral ground-glass opacities. Microbiologic evaluation, including β-D-glucan and *Pneumocystis jirovecii* PCR, was negative, and clinically suspected drug-induced interstitial pneumonitis was diagnosed. Anticancer therapy was immediately discontinued, and two courses of high-dose intravenous methylprednisolone pulse therapy were administered, followed by oral corticosteroid tapering. Despite early intervention, radiologic abnormalities progressed, and hypoxemia worsened, ultimately resulting in fatal respiratory failure. Notably, serum PSA declined to 0.07 ng/mL and remained suppressed throughout the period of pulmonary deterioration, indicating a sustained oncologic response despite lethal toxicity. This case underscores that life-threatening interstitial lung disease may occur during docetaxel-based triplet therapy even in the absence of preexisting pulmonary risk factors. Early imaging for subtle respiratory symptoms and prompt multidisciplinary management are essential to mitigate potentially fatal outcomes.

## Introduction

Triplet therapy combining androgen deprivation therapy (ADT), an androgen receptor-axis targeted agent (ARAT), and docetaxel has become a standard treatment for metastatic hormone-sensitive prostate cancer (mHSPC), offering significant survival benefit as demonstrated in the ARASENS and PEACE-1 trials [1,2]. As this intensified strategy becomes increasingly adopted in routine practice, particularly among elderly patients, attention to cumulative and unexpected toxicities has become important.

Although triplet therapy has shown a favorable safety profile in clinical trials, real-world safety data remain limited. In pivotal studies, interstitial lung disease (ILD) was reported in fewer than 1% of patients receiving docetaxel-based regimens, and severe pulmonary toxicity was rare [3-5]. However, clinical trials may underestimate uncommon but serious adverse events, especially in older populations or those with subclinical vulnerability.

Pulmonary adverse events, particularly interstitial pneumonitis, have been described with taxanes and newer hormonal agents [6-9]. Most reported cases were reversible following treatment discontinuation and corticosteroid therapy. Fatal ILD during combination therapy, particularly in patients without prior lung disease or thoracic irradiation, has rarely been documented. In large phase III trials, clinically significant ILD associated with ADT or ARAT agents has been reported in less than 1% of patients, and fatal pulmonary toxicity remains exceedingly rare. Degarelix has not been strongly associated with pulmonary toxicity, while darolutamide demonstrated respiratory adverse event rates comparable to the placebo in pivotal trials.

We describe a fatal case of drug-induced ILD occurring during triplet therapy in a patient with low-volume mHSPC, emphasizing that life-threatening pulmonary toxicity may occur even in clinically stable individuals without recognized risk factors.

## Case presentation

A 79-year-old man with mHSPC presented with low-grade fever and exertional dyspnea after the fifth cycle of docetaxel. On admission, oxygen saturation was 91% on room air with bilateral fine crackles.

The patient was diagnosed with low-volume mHSPC according to the CHAARTED criteria. Staging imaging revealed multiple pelvic lymph node metastases and a solitary bone metastasis in the S1 sacral vertebra, without evidence of visceral involvement. According to the CHAARTED definition, high-volume disease requires visceral metastasis or ≥4 bone metastases with at least one beyond the vertebral bodies and pelvis. As neither criterion was met, the disease was classified as low-volume. Prostate biopsy revealed Gleason 5 + 5 = 10 adenocarcinoma involving all cores, and the baseline prostate adenocarcinoma (PSA) was 613 ng/mL. The patient had no history of smoking, lung disease, or thoracic radiation.

**Figure 1:**
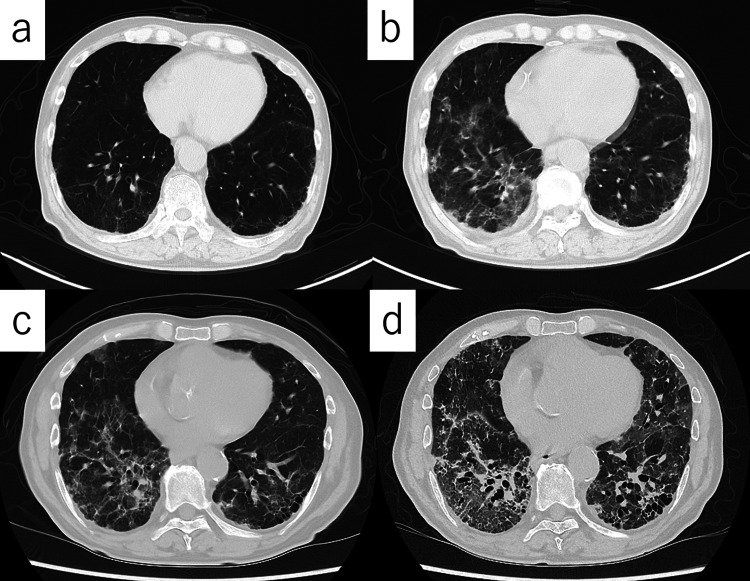
Radiologic course of drug-induced interstitial pneumonitis. Sequential chest CT scans showing the rapid progression of diffuse, non-segmental ground-glass opacities despite corticosteroid therapy (a) Baseline CT prior to symptom onset. (b) Day 0 (on hospital admission): newly developed bilateral ground-glass opacities. (c) Day 11 (after the first course of corticosteroid pulse therapy): no radiologic improvement observed. (d) Day 24 (after the second pulse therapy): bilateral consolidation and traction bronchiectasis further progressed, consistent with fatal drug-induced ILD. CT: computed tomography, ILD: interstitial lung disease

Triplet therapy was initiated, consisting of degarelix, darolutamide (1200 mg/day), and docetaxel (70 mg/m² every three weeks). After completing five cycles of docetaxel, he developed a low-grade fever and exertional dyspnea. Chest CT demonstrated diffuse, non-segmental ground-glass opacities in both lungs (Figure 1a-1b).

The patient had no history of autoimmune disease or chronic cardiac or renal disease and was not taking any medications known to be associated with pulmonary toxicity.

Differential diagnosis, investigations, and treatment

Differential diagnoses included infectious pneumonia (bacterial, viral, including SARS-CoV-2, fungal, and *Pneumocystis jirovecii*), cardiogenic pulmonary edema, pulmonary embolism, autoimmune-related ILD, and lymphangitic carcinomatosis. PCR for β-D-glucan and *Pneumocystis jirovecii* were negative. Brain natriuretic peptide levels were within normal limits, and transthoracic echocardiography demonstrated trivial mitral regurgitation and mild-to-moderate tricuspid regurgitation, with an estimated right ventricular systolic pressure of 51 mmHg, suggesting possible pulmonary hypertension but no evidence of left-sided heart failure. There were no clinical signs of volume overload, such as peripheral edema or jugular venous distension. Autoimmune workup was performed and was largely unremarkable. Matrix metalloproteinase-5 (MMP-5) and surfactant protein D levels were elevated (556.9 ng/mL and 559.8 ng/mL, respectively), consistent with interstitial lung injury, while other serologic markers were within normal limits. Laboratory evaluation on admission revealed elevated inflammatory markers, including C-reactive protein 11.46 mg/dL and lactate dehydrogenase 310 U/L.

Blood cultures, viral PCR testing, including SARS-CoV-2, and routine bacterial cultures were negative, and clinically suspected drug-induced interstitial pneumonitis was diagnosed. High-dose intravenous methylprednisolone (1000 mg/day for 3 days) was started, followed by tapering oral corticosteroids. The transient improvement referred specifically to temporary stabilization of oxygen saturation under supplemental oxygen and a reduction in inflammatory markers (CRP and LDH); however, radiologic abnormalities did not significantly improve. A second course of methylprednisolone pulse therapy (1000 mg/day for three days) was administered, again with a temporary response. A second pulse course was administered due to radiologic progression and worsening oxygen requirement despite initial therapy. Oxygen supplementation was initiated at 2 L/min via nasal cannula on admission and maintained until hospital day 40. The oxygen requirement increased to 3 L/min on day 41 and further escalated to 5 L/min via oxygen mask on day 59. During meals, up to 8 L/min via oxymizer was required to maintain oxygen saturation. Two days prior to death, high-flow nasal cannula therapy was initiated due to worsening hypoxemia. Arterial blood gas analysis was not performed because the patient was already receiving continuous supplemental oxygen, and oxygenation was closely monitored using pulse oximetry. Given the progressive clinical deterioration and limited impact on management strategy, invasive arterial sampling was not prioritized. The decision was made in agreement with the pulmonology team. Bronchoscopy with bronchoalveolar lavage was not performed based on pulmonology consultation due to progressive respiratory compromise, advanced age, and concern for procedural risk under escalating oxygen support. Given the clinical instability and high suspicion of drug-induced lung injury after exclusion of major infectious etiologies, empiric corticosteroid therapy was prioritized over invasive diagnostic procedures.

Despite intensive corticosteroid therapy and supportive care, hypoxemia progressed, and the patient died of respiratory failure due to ILD. Throughout the illness, his PSA level declined to 0.07 ng/mL and remained suppressed, indicating sustained oncologic control despite fatal pulmonary toxicity. Adverse event grading was assessed according to the Common Terminology Criteria for Adverse Events (CTCAE) version 5.0. The ILD observed in this case corresponded to Grade 5 (death related to adverse event). The corticosteroid regimen consisted of intravenous methylprednisolone 1000 mg/day for three consecutive days, followed by oral prednisolone 1 mg/kg/day tapered over four weeks. Despite early initiation, respiratory failure progressed, and long-term oxygen therapy was required until death. No elevation in β-D-glucan or detection of *Pneumocystis jirovecii* by PCR was noted, supporting a non-infectious drug-induced ILD.

Chest CT demonstrated diffuse, non-segmental ground-glass opacities in both lungs (Figure 1a-1b). Despite high‐dose corticosteroid pulse therapy, radiologic abnormalities gradually worsened, as shown on follow-up CT (Figure 1c-1d). The overall clinical timeline and sequence of therapeutic interventions are illustrated in Figure 3.

**Figure 2:**
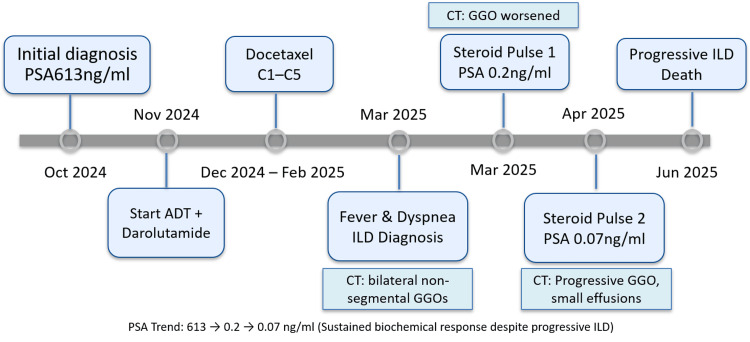
Graphical clinical timeline of triplet therapy and fatal interstitial lung disease A schematic timeline summarizing the treatment course and major clinical events. Following initiation of ADT plus darolutamide and five cycles of docetaxel, the patient developed drug-induced ILD in March 2025. Despite two courses of high-dose corticosteroids and sustained PSA suppression (613 → 0.2 → 0.07 ng/mL), pulmonary injury progressed and resulted in death in June 2025. ADT: androgen deprivation therapy, ILD: interstitial lung disease, CT: computed tomography, GGO: ground-glass opacity Image Credit: Authors using Microsoft PowerPoint (Microsoft Corp., Redmond, WA, USA)

**Figure 3:**
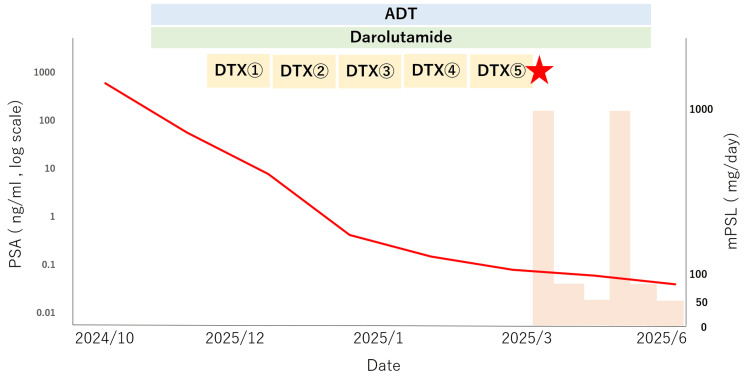
Clinical course during triplet therapy Showing serum PSA levels (log scale), corticosteroid dosage (pink area, right axis), and treatment schedule. ADT, darolutamide, and DTX (①–⑤) cycles are indicated on the timeline. The red star (★) marks the onset of interstitial lung disease, which occurred shortly after the fifth DTX cycle. Although PSA levels remained markedly suppressed, tapering of corticosteroids failed to prevent progressive respiratory failure. PSA: prostate adenocarcinoma, ADT: androgen deprivation therapy, DTX: docetaxel Image Credit: Authors using Microsoft Excel and Microsoft PowerPoint (Microsoft Corp., Redmond, WA, USA)

Fatal interstitial pneumonitis can occur during triplet therapy for mHSPC even in the absence of predisposing factors. The concurrent use of ARAT and docetaxel under androgen suppression may potentiate pulmonary toxicity. Of note, serum PSA remained deeply suppressed throughout the course of illness, indicating sustained oncologic control despite progressive respiratory failure (Figure 3).

## Discussion

Triplet therapy combining ADT, an ARAT, and docetaxel has become an established first-line regimen for mHSPC. Large randomized trials such as ARASENS and PEACE-1 demonstrated significant improvements in overall survival, particularly among patients with high-volume disease [1,2]. According to current international guidelines and pivotal phase III trials, treatment intensification with ADT combined with ARAT agents and/or docetaxel is recommended for eligible patients with mHSPC regardless of disease volume. Although the magnitude of benefit may be more pronounced in high-volume disease, survival advantages have also been demonstrated in low-volume cohorts, supporting the use of intensified regimens in appropriately selected patients. However, the increasing use of this multimodal approach has also raised awareness of overlapping or cumulative toxicities that may emerge when combining systemic therapies [3-5]. Among these, pulmonary complications such as interstitial pneumonitis are rare but potentially fatal adverse events.

ILD associated with anticancer therapy can arise from various mechanisms, including direct drug toxicity, immune-mediated injury, or interactions between agents. In prostate cancer, several drugs have been implicated, including docetaxel, cabazitaxel, apalutamide, and the PARP inhibitor olaparib [6,9,10-12]. Most reported cases were reversible after discontinuation of the drug and corticosteroid treatment [9-12]. Fatal outcomes have been uncommon, and when they occurred, patients often had prior thoracic irradiation or preexisting pulmonary disease [12,13]. In contrast, our patient had no baseline risk factors and developed rapidly progressive ILD after five cycles of triplet therapy, culminating in respiratory failure despite two courses of high-dose corticosteroids. To our knowledge, this represents one of the first fatal cases of ILD occurring during triplet therapy for mHSPC without prior pulmonary comorbidities.

Possible mechanisms of lung injury

The etiology of ILD in this setting is likely multifactorial. Docetaxel-induced interstitial pneumonitis has been recognized since its early use in prostate and breast cancers. Proposed mechanisms include direct cytotoxic injury to alveolar epithelial cells and an aberrant immune response leading to diffuse alveolar damage (DAD) [6,12]. ARAT agents such as apalutamide and enzalutamide have also been associated with immune-mediated pulmonary inflammation, possibly through cytokine upregulation or altered T-cell signaling [9,10]. In triplet regimens, the concurrent administration of ARATs and cytotoxic chemotherapy may exacerbate subclinical alveolar injury, lowering the threshold for clinically overt ILD.

In our patient, the temporal association between docetaxel exposure and onset of respiratory symptoms, together with negative microbiologic studies and radiologic findings, strongly suggested a drug-related etiology. It is plausible that cumulative docetaxel exposure triggered alveolar epithelial damage, while simultaneous ARAT and ADT exposure modulated immune repair mechanisms, leading to uncontrolled inflammation. Additionally, age-related vulnerability and androgen axis suppression may impair tissue resilience. These factors collectively contributed to irreversible pulmonary injury despite early corticosteroid intervention. Radiologic findings were most compatible with a DAD-like pattern rather than organizing pneumonia, given the rapid progression and development of traction bronchiectasis.

Diagnostic challenges

Diagnosing drug-induced ILD in prostate cancer patients is challenging because symptoms such as fatigue or dyspnea may initially be attributed to treatment-related malaise or anemia. Furthermore, docetaxel and ARATs are rarely associated with respiratory toxicity in clinical trials, with reported ILD incidence <1% [3-5]. Consequently, clinicians may underestimate early pulmonary signs. In our case, the patient presented with only a low-grade fever and mild dyspnea after the fifth docetaxel cycle, yet imaging revealed widespread ground-glass opacities. Prompt recognition, infection exclusion, and immediate initiation of corticosteroids were appropriate, but the disease still progressed rapidly. This emphasizes that once ILD is suspected during triplet therapy, treatment interruption and early multidisciplinary consultation (oncology, pulmonology, and radiology) are essential.

Pathologic confirmation was not obtained. Therefore, the diagnosis remains clinically presumptive; however, the temporal association with docetaxel exposure, absence of infectious or cardiogenic causes, and radiologic progression consistent with interstitial lung injury strongly support a drug-related etiology.

Comparison with previous reports

A review of verified reports (Table 1) shows that most cases of prostate cancer-related ILD involved single-agent therapy and recovered after corticosteroids [9-12]. Kirishima et al. described an apalutamide-induced ILD that resolved with steroid therapy [10]. Yanai et al. reported cabazitaxel-related pneumonitis occurring after thoracic irradiation [9]. Kaitsumaru et al. documented olaparib-induced ILD in a BRCA1-mutated neuroendocrine carcinoma that improved after drug withdrawal [11]. A docetaxel-related case reported in 2022 also recovered after pulse steroids [12]. In contrast, our case was fatal despite aggressive corticosteroid therapy, suggesting a possible additive or synergistic pulmonary toxicity when combining docetaxel and ARAT under androgen suppression.

**Table 1:** Verified reports of drug‐induced ILD during prostate cancer treatment Most previously reported cases involved single-agent therapy with apalutamide, cabazitaxel, olaparib, or docetaxel, and all recovered after corticosteroid treatment. The present case represents, to our knowledge, the first fatal ILD associated with triplet therapy (degarelix + darolutamide + docetaxel) for mHSPC, underscoring potential additive pulmonary toxicity even under adequate disease control. CRPC: castration-resistant prostate cancer, RT: radiotherapy, mPSL: methylprednisolone, NEPC: neuroendocrine prostate cancer, BRCA1-mut: BRCA1-mutated, PCa: prostate cancer, mHSPC: metastatic hormone-sensitive prostate cancer, ILD: interstitial lung disease

Author (year)	Agent(s)	Setting	Prior lung disease/thoracic RT	Onset timing	Management	Outcome
Yanai et al. 2019 [9]	Cabazitaxel	CRPC	Post-RT	During treatment	Steroids	Improved
Kirishima et al. 2022 [10]	Apalutamide	CRPC	None	Within 3 months	mPSL → taper	Recovered
Kaitsumaru et al. 2023 [11]	Olaparib	NEPC/BRCA1-mut	None	Weeks after initiation	Drug withdrawal ± steroids	Improved
Hettiarachchi et al. 2021 [12]	Docetaxel	Advanced PCa	None	2 weeks after 6 cycles	Steroids	Recovered
Present case (2025)	Triplet (degarelix + darolutamide + docetaxel)	mHSPC (low-volume)	None	After 5 cycles	Two steroid pulses → taper	Fatal

Management of drug-induced ILD relies on immediate discontinuation of the causative agents and administration of systemic corticosteroids. Pulse therapy (methylprednisolone 500-1000 mg/day for 3 days) is recommended for severe or rapidly progressive cases, followed by gradual tapering [13,14]. However, optimal duration and re-challenge strategies remain unclear in prostate cancer, where treatment options are limited. In metastatic prostate cancer, continuation of effective systemic therapy is often essential for disease control, limiting opportunities to withhold or modify treatment compared with non-malignant ILD. In our case, despite two steroid pulses and prolonged tapering, pulmonary infiltrates continued to worsen. This suggests that once DAD becomes established, even high-dose corticosteroids may not reverse the fibrotic cascade. Additional immunosuppressive agents such as cyclophosphamide or mycophenolate mofetil have been used in other drug-induced ILDs but lack evidence in this context [13]. In addition to corticosteroid therapy, supportive management included early initiation of high-flow nasal cannula oxygen therapy under ICU-level monitoring, followed by noninvasive positive-pressure ventilation for refractory hypoxemia. However, oxygenation gradually worsened despite maximal supportive care. Second-line immunosuppressive agents or antifibrotic therapy, such as nintedanib, was not administered because of rapid clinical deterioration and lack of established evidence supporting their use in acute drug-induced ILD in prostate cancer.

Clinicians should maintain a high index of suspicion for ILD in patients who develop new-onset fever, cough, or dyspnea during or after docetaxel-based triplet therapy [6,14]. In such cases, prompt chest CT imaging and careful exclusion of major infectious and cardiac causes are essential steps in the diagnostic evaluation [14,15]. When drug-induced ILD is suspected, the suspected agents should be discontinued immediately, and high-dose corticosteroid pulse therapy should be initiated without delay [13,14]. In rapidly progressive cases, escalating oxygen support may also be necessary to manage respiratory compromise [13]. Re-challenge with the offending agents is generally discouraged, particularly in patients with suspected DAD-pattern ILD [13,16].

Given that PSA remained deeply suppressed until death, it is reasonable to assume that tumor control was maintained throughout the pulmonary decline. This paradox highlights a key clinical dilemma: patients may achieve oncologic benefit from triplet therapy while simultaneously developing life-threatening toxicity. Thus, close respiratory monitoring is warranted not only at treatment initiation but also during maintenance or tapering phases, particularly in elderly patients. Baseline pulmonary evaluation and early imaging for any new respiratory symptoms should be considered standard practice in patients receiving docetaxel-based triplet therapy. This case provides actionable monitoring cues for respiratory toxicity during docetaxel-based triplet therapy and a stepwise response protocol that can be replicated in routine care.

Lessons learned and clinical implications

This case underscores several important clinical lessons. First, drug-induced ILD should remain a differential diagnosis whenever respiratory symptoms arise during triplet therapy, even in patients without prior lung disease. Second, the timing of onset, after several docetaxel cycles, suggests that cumulative toxicity may play a role; clinicians should maintain vigilance beyond the early cycles. Third, the absence of infection and lack of response to high-dose corticosteroids may indicate irreversible lung injury, emphasizing the importance of prevention and early recognition over reactive management. Finally, this case highlights the need for post-marketing pharmacovigilance and registry data on pulmonary toxicity during combination therapy in mHSPC, as clinical trials may underestimate the true incidence in real-world settings. To our knowledge, this represents one of the very few reported cases of fatal ILD occurring during triplet therapy with degarelix, darolutamide, and docetaxel in mHSPC. Previous reports have described reversible ILD associated with cabazitaxel [9], enzalutamide [10], or olaparib [11], but fatal courses remain exceptional [12,13]. This case underscores that even in the absence of prior lung disease or radiation, vigilant monitoring for respiratory symptoms is essential during combined androgen- and taxane-based regimens.

## Conclusions

This case underscores that fatal ILD can occur during docetaxel-based triplet therapy even in patients without preexisting pulmonary risk factors. Although triplet therapy provides substantial oncologic benefit, clinicians must remain vigilant for rare but life-threatening pulmonary toxicity. Early imaging, prompt discontinuation of all antineoplastic agents, and timely corticosteroid administration are essential when new respiratory symptoms arise. Structured respiratory surveillance may be warranted, particularly in elderly patients receiving intensified systemic therapy.
